# Carbon Texture Formation on the Surface of Titanium Alloy Grade 5 (Ti–6Al–4V) During Finishing with Abrasive Films

**DOI:** 10.3390/molecules30030514

**Published:** 2025-01-23

**Authors:** Katarzyna Tandecka, Wojciech Kacalak, Dominika Panfil-Pryka, Michał Wieczorowski, Thomas G. Mathia

**Affiliations:** 1 Department of Engineering and Informatics Systems, Faculty of Mechanical Engineering and Energy, Koszalin University of Technology, 75-620 Koszalin, Poland; wojciech.kacalak@tu.koszalin.pl; 2 Faculty of Mechanical Engineering, Institute of Applied Mechanics, Poznan University of Technology, 60-965 Poznan, Poland; dominika.panfil-pryka@put.poznan.pl (D.P.-P.); michal.wieczorowski@put.poznan.pl (M.W.); 3 Laboratoire de Tribologie et Dynamique des Systemes (LTDS), Ecole Centrale de Lyon, Centre National de la Recherche Scientifique, 69134 Lyon, France

**Keywords:** microfinishing, carbon texture, graphite-coated abrasive films, titanium alloy

## Abstract

This research explored the formation and effects of carbon layers on Grade 5 titanium alloy (Ti–6Al–4V) surfaces during a microfinishing process using both traditional abrasive films and graphite-coated abrasive films. The study tried to appraise the effect of using graphite-coated films in the microfinishing process concerning surface roughness. Microfinishing with an abrasive film impregnated with diamond particles and an additional coating of graphite was performed to minimize surface roughness and enhance the overall performance of the surface. As a result, it was shown that after processing, the uniform carbon texture formed by the graphite-coated film significantly improved the lubricating and thermal properties. Energy dispersive spectroscopy (EDS) analysis confirmed the homogeneity of carbon distribution over the whole treated surface. Moreover, the graphite-coated films enabled us to obtain smoother surfaces with improved tribological properties. The study therefore concluded that the inclusion of graphite in the abrasive films is necessary for effecting surface modification in light of considerable improvements in surface quality and performance, especially where the wear needs to be reduced and the integrity of the surface maximized.

## 1. Introduction

Several techniques are used to form carbon layers, all of which avail advanced methods for different usages. For instance, one of the techniques is the electron-beam partial melting of carbon fibers, a method used in forming high-carbon layers on low-carbon steel. This can result in a carbon layer that is 3 mm thick with a 2.2% carbon content, usable in some industrial applications for its better surface properties [1]. Another method for the preparation of nanostructured carbon layers doped with cobalt is by the dehydrochlorination of polyvinyl chloride in the presence of nitrate, followed by heat treatment. This technique yields continuous carbon coatings with unique advantages for materials that require strong, uniform coatings [2]. Other extensively studied processes include carbon deposition over catalysts, as in the case of deposition over Ni/Al_2_O_3_ methanation catalysts, where it was shown that both the morphology and quantity of the carbon deposits that formed on the surface of a catalyst could be affected by the temperature and H_2_/CO ratio.

Among the various synthesis methods, chemical vapor deposition (CVD) plays a significant role in the preparation of carbon layers including its variants, plasma-enhanced chemical vapor deposition (PECVD), arc discharge, and low–temperature CVD. All of these techniques are diverse in terms of the conditions under which the carbon layers have been produced, thereby offering immense applicative scope and different outcomes from one procedure to the other [3]. Of course, the low-temperature CVD method has attracted a lot of attention in light of its possibility to yield carbon nanostructures, which may find potential applications in semiconductor devices. The technique presented allows for the precise layer-by-layer deposition of carbon atoms, and thus should be highly welcome in microelectronics and other hi-tech areas where high-quality material growth over large areas is of crucial importance [4]. In the high- and low-temperature versions, CVD has a particular advantage in carbon layer formation, and has been applied not only in the preparation of carbon nanotubes and nanofibers, but also with diamond and graphene layers, with huge applications in different technological fields [5].

Among the conventional methods for carbon material synthesis, PECVD is popular due to the low temperature it requires for synthesizing materials and thus can grow vertical structures such as carbon nanotubes (CNTs) and carbon nanowalls (CNWs) [6]. It has also been shown that the morphology of the carbon layers can be influenced by changing the plasma conditions, and therefore their performance in specific applications such as wear resistance, lubrication, and surface modification [7]. These carbon coatings are known to reduce friction and improve mechanical durability, which are critical for many industrial uses where surface wear and friction reduction are crucial [8].

Because of the properties they possess, carbon layers are widely used in many areas of human activity starting from biomedical engineering and ending with mechanical ones. Generally, these layers are deposited on engineering and biomedical materials, nanostructures, and carbon nanotubes [9]. The CVD method allows for several types of materials to be deposited, among which carbon is included, in a form such as nanofibers, nanotubes, diamond, graphene, and even tungsten and titanium nitride [5]. Carbon layers synthesized by the CVD method are widely applied, starting from the structure of optoelectronic devices on gallium arsenide up to the implementation of cutting tools, biological sensors, infrared imagining technologies, etc. [10]. This approach allows for the elaboration of high-quality surface layers required in many applications including ternary cathode-based cylindrical batteries [11].

One of the big advantages of CVD is that carbon coatings can be deposited on surfaces locally, even on very controlled industrial surfaces. For in situ analysis in the study of nucleation and the growth of thin film materials, scanning probe microscopy is used. The material in question is formed from the condensation of carbon in a hydrogen/methane gas mixture, activated by the “hot-filament” method [7]. Applications pertaining to CVD methods can entail a range of techniques in high temperature, plasma-enhanced, low pressure, and laser ablation, all operating at different temperature/pressure conditions. These have made it possible to prepare carbon materials with unique properties that improve the mechanical and thermal properties, thus making it suitable for industries that have uses requiring low-friction and high-resistance wear properties such as the aerospace, biomedical, and electronic industries [12].

A number of techniques, like the use of titanium interlayers, continue to optimize carbon coatings on surfaces for better adhesion. These layers are characterized by excellent sliding properties, which are particularly valuable in the case where the use of process fluids cannot be afforded. Because of this, they are very suitable for mechanical engineering and medical applications [13]. Furthermore, carbon films are resistant to abrasions, chemical wear, and possess biocompatibility, which extends their application in implants and orthopedics [14,15]. Their properties vary from highly elastic to resistant thermal shock, hence, their applications vary across a wide range of industries.

Particularly, diamond-like carbon (DLC) films have been widely used in cutting and machining non-ferrous metals and as non-metallic materials for improving the mechanical and thermal properties [16,17]. These thin layers have excellent wear resistance and low friction, which is very important for industries requiring precision and durable components. In this context, the addition of metals like titanium to carbon films results in a dramatic increase in adhesion between the carbon layer and the substrate, and increases the elasticity and other mechanical properties of the material [18,19]. Large numbers of multilayer thin carbon films are applied as protective coatings in various applications, from electronic packaging to structural composites [20]. These films help in thermal protection, abrasion, and corrosion resistance, all of which are very important for improving durability and performance in very harsh environments [21].

**Figure 1:**
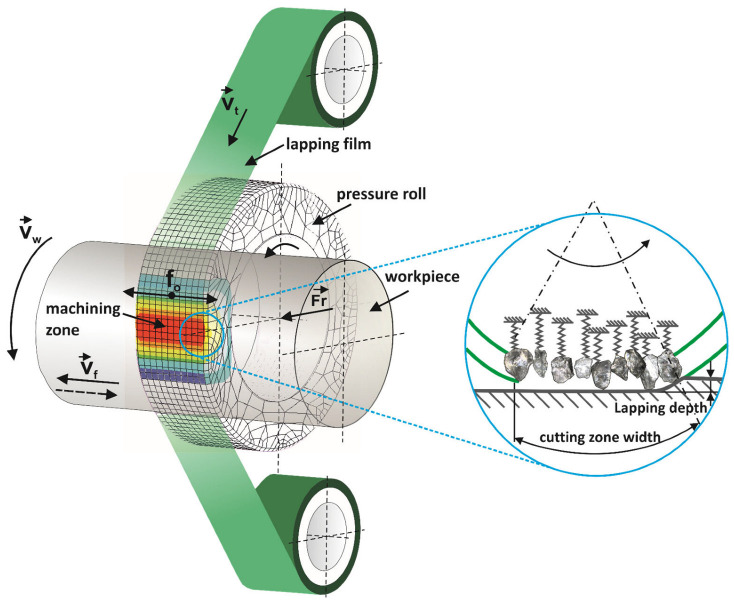
Diagram of the microfinishing process using abrasive films [35].

Microfinishing processes belong to a class of surface treatment techniques that have gained recognition for their ability to dramatically reduce the surface roughness down to very fine or extremely smooth surfaces [22,23,24]. In superfinishing, the sequential treatment consists of microabrasive rolls of increasingly finer grain size starting from 30 μm down to 0.5 μm by successive tool changes [25,26] (Figure 1). This technique is usually adopted for rotating workpieces but can be adapted for other geometrical forms of the surface [27]. The abrasive layer is generally made of materials like noble corundum, synthetic diamond, or silicon carbide [28,29,30,31,32,33,34]. All of these processes are optimized for the best utilization of the tools, since the abrasive film travels rather slowly compared to the feed rate of the workpiece, so high-quality results can be guaranteed.

**Figure 2:**
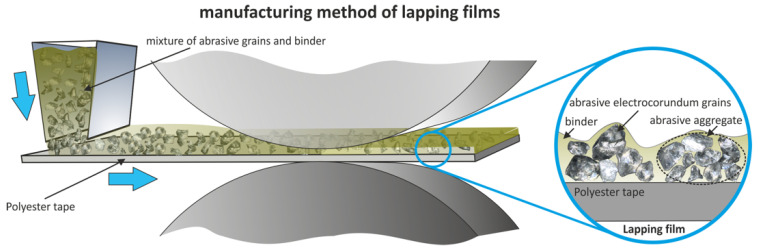
Production process of lapping-type abrasive films [35].

Lapping films are specialized abrasives in which a layer of abrasive particles is bonded to a flexible polyester tape with an appropriate binder (Figure 2). The products are manufactured by the careful mixing of abrasive grains, usually electrocorrundum, in a suitable binder that is spread onto the polyester substrate [29,36,37]. This will provide a high-performance product for precision finishing and polishing in aerospace, medical, automotive, and electronics [38,39,40]. Most manufacturing processes commence with the selection of the abrasive particles, whose size and hardness differ depending on the application [41,42,43]. These abrasives are combined with a binder material, normally polymer resin, into a slurry. Next comes the coating, when the slurry is applied onto the polyester backing tape [42,44,45,46]. This is achieved by the production of a properly adhering abrasive layer onto the tape, so that the product lasts long for perfect material removal [47,48,49,50].

The main characteristic of lapping films concerning performance is a fine, smooth finish over various types of surfaces. These are available all the way from coarse to ultra-fine in grit size, which extend its use into applications where other abrasives do not or will not work. Coarse grits are useful in initial rough polishing; finer grits for finishing work to high-precision surface finishes [45,51,52]. Moreover, the flexibility of the polyester backing means that these films will follow different surface contours and are therefore particularly useful for processing flat and irregular shapes. Generally, lapping films have an excellent consistent cutting action together with a uniform distribution of abrasives, causing less surface roughness as well as leading to improvements in the dimensional accuracy [36,48]. Such a combination of fine abrasives on flexible substrates allows for finer process control, which results in greater superior performance compared with traditional abrasive papers. Additionally, the application of lapping films in applications where precision and surface integrity are at stake, like the manufacture of optical components, semiconductor wafers, or medical devices, underscores their importance for quality in industries.

Further improvement in the surface characteristics, such as a reduction in roughness and an improvement in tribological performance, could possibly be carried out with the application of carbon coatings on the workpiece surface. This paper discusses an innovative process chain that links superfinishing to the deposition of carbon layers, a rising technology that integrates finishing and carbon coating into one process. The technique presented in this paper has been patented by the authors, and thus could potentially improve the efficiency and effectiveness in many cases of surface treatments.

This research aims to establish the feasibility and effectiveness of depositing carbon films onto surfaces using graphite-impregnated lapping films. In fact, all of the integrated processes reduced the roughness of the surface and enhanced the durability and wear resistance of the materials treated. Previous studies have focused on either the surface finishing methods or carbon coating techniques in isolation, but not together, as in this study. Much of the research in surface engineering and tribology has proven that carbon-based coatings reduce friction and wear. However, their integration with microfinishing techniques is very recent, with the potential to transform the face of surface treatment procedures.

The results obtained in this work demonstrate that graphite-filled lapping films can deposit homogeneous carbon layers onto various surfaces, increasing the quality and lifetime of applications with large areas. In particular, the application of ultrathin graphite layers onto polished surfaces is of benefit to systems that stay at rest for long times but must still deliver the optimum performance without further servicing. Such systems could be kinematic joints with limited access, those used in military technologies, or applications running under vacuum conditions and at low temperatures.

In recent years, a lot of attention has focused on the growth of carbon layers on surfaces in order to improve the tribological properties of materials; in particular, for improving the wear resistance and reducing the friction of titanium alloys, extensively used in the aerospace, medical, and automotive industries [12,18,53,54,55]. Microfinishing is one of the methods used in the improvement of surface quality and is noted to have high potential in attaining such goals. The role of carbon coatings, especially of graphite origin, in the microfinishing process, however, is still under active research.

The primary purpose of the current research was to investigate the formation and uniformity of carbon layers on the surface of titanium alloy Grade 5 (Ti–6Al–4V) during the microfinishing process. The study focused on the influence of graphite-coated abrasive films on the surface characteristics, mainly roughness. The present work aimed to show the possibility of integrating carbon deposition with microfinishing as a novel surface engineering method, which would offer significant improvements in wear resistance and surface integrity. The present research work investigated the process of carbon layer formation on the surface of Ti–6Al–4V titanium alloy in microfinishing with conventional and graphite-coated abrasive films. This was the basic study to understand how the use of graphite could influence surface characteristics such as roughness. This investigation aimed at testing the hypotheses proposed on the homogeneity of the carbon texture. The present study will attempt to investigate the influence of graphite coatings on the surface of titanium alloys with an emphasis on the implications for surface roughness. The present research will, therefore, provide important insights for applications where better surface properties are of great importance. The main findings arising from this research will increase the knowledge on surface modification techniques that could lead to future innovations in the manufacturing of components that require improved wear resistance and lower friction. Modified abrasive films with a graphite coating offer a low-cost approach to achieve superior surface properties. Results were consistent, minimizing rework or secondary processing. In addition, the deposition of carbon layers combined with the microfinishing process eliminated the need for separate coating and smoothing operations, which further streamlines efficiency while lowering the total production costs.

## 2. Results and Discussion

This section presents the results obtained in experiments performed on Grade 5 titanium alloy (Ti–6Al–4V) samples subjected to a novel microfinishing process. The process exploits abrasive films impregnated with diamond particles of a nominal 0.5 μm grain size, further modified by coating with a layer of graphite. The results show the influence of such an approach on the surface roughness and material composition.

**Figure 3:**
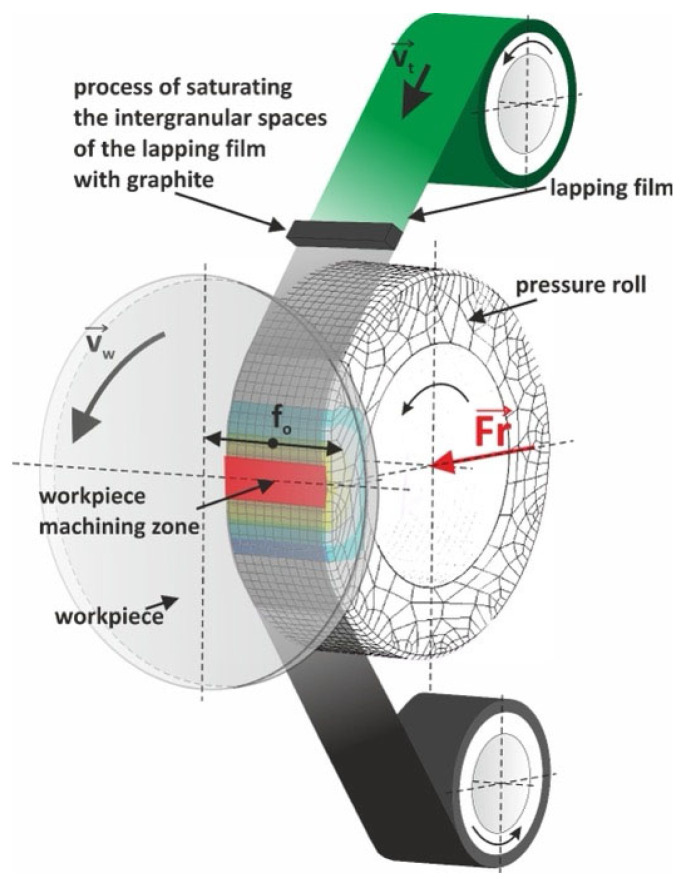
The process of applying a graphite layer to the abrasive film prior to the microfinishing process [56].

Microfinishing was performed in order to increase the polish of the surface of the titanium alloy and obtain the uniform application of a carbon layer. The carbon layer was produced by the combined action of the pressure roll, abrasive particles in the film, and relative motion between the workpiece and film (Figure 3). Such an interaction led to a dramatic decrease in the surface roughness and the formation of a uniform surface layer.

**Figure 4:**
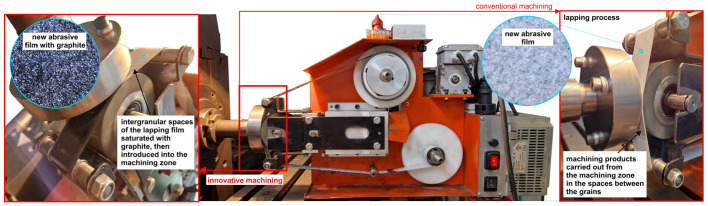
Test stand for the microfinishing process with the microfinishing attachment and abrasive film coated with a graphite layer (**left**) and conventional machining (**right**).

Investigation into the microfinishing process was conducted using a microfinishing attachment (GW–1) that can be fitted to standard machine tools (Figure 4). The study was primarily on the microfinishing process, with an abrasive film coated with graphite representing a coherent approach in surface refinement and the deposition of a carbon layer on the machined surfaces. Samples that had been prepared by preliminary machining in an identical way underwent conventional processing to make the results comparable.

The graphite coating in the microfinishing process provided a smoother workflow and improved the surface characteristics while simultaneously reducing the roughness and enhancing the tribological performance. The technique is useful in using the flexibility of the microfinishing attachments and can be further adopted for diverse machining environments and materials. Comparisons provided good insights into the efficiency and effectiveness of the integrated microfinishing process over conventional methods.

### 2.1. Analysis of the Carbon Layer

**Figure 5:**
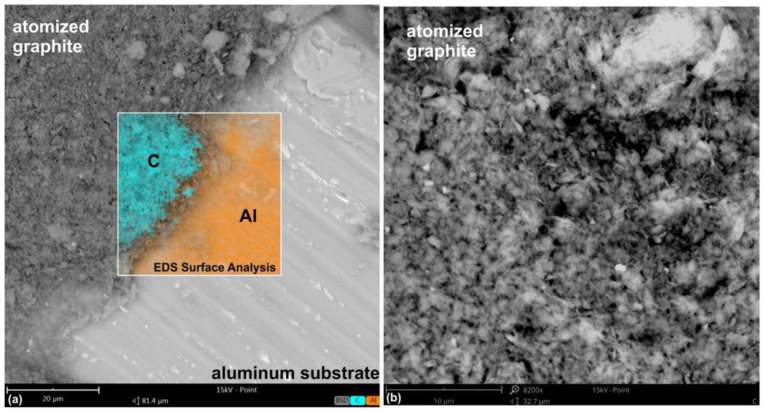
SEM image of a graphite-coated aluminum substrate with surface EDS elemental composition analysis (**a**) and the zoomed-in area of atomized graphite (**b**).

In the initial stages of the research, a layer of carbon in the form of atomized graphite was deposited over an aluminum substrate; this decision was made according to its property of not containing carbon within its own chemical formula, which would allow for a detailed view of the chemical composition of the laid carbon layer. The carbon layer consisted of graphite flakes. Its chemical composition, as determined by energy dispersive X-ray spectroscopy (EDS) analysis, contained only carbon at 100% (Figure 5). With that in mind, the atomized graphite ensured that there was a homogeneous, well-adhered layer, which is important for the evaluation of its properties. This structure of the graphite flakes further increases their lubricating property and makes them especially valuable for many applications where surface properties like reduced friction, an augmented level of thermal conductivity, and excellent electrical conductivity come into play. The EDS confirmed the uniformity and purity of the carbon layer, showing that no impurities or irregularities occurred within the deposition process.

**Figure 6:**
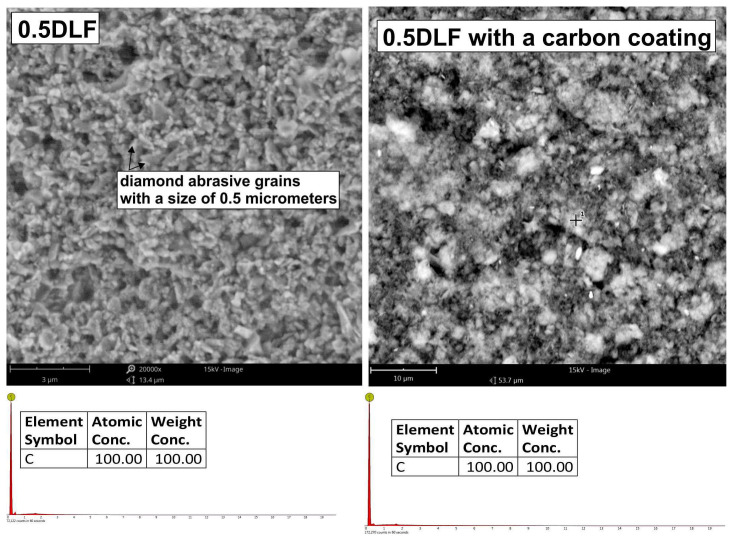
SEM images of a new diamond-coated abrasive film with a nominal grain size of 0.5 μm (**left** side) and the same abrasive film coated with a graphite layer.

**Figure 7:**
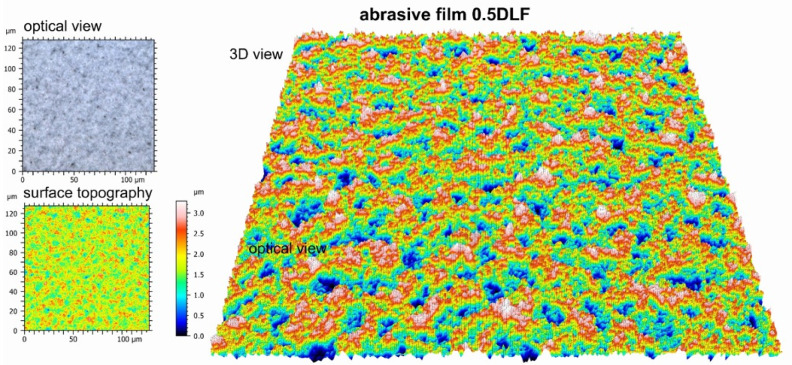
Topographic and optical images of a diamond-coated abrasive film with a nominal grain size of 0.5 μm.

**Figure 8:**
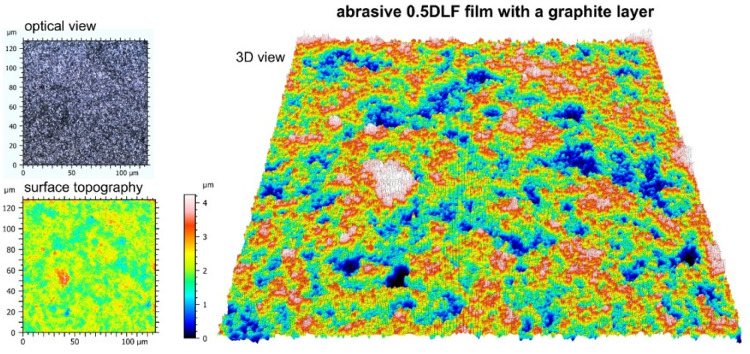
Topographic and optical images of a diamond-coated abrasive film with a nominal grain size of 0.5 μm coated with a graphite layer.

Chemical compositions for the surface of the standard 0.5 DLF (diamond lapping film)-abrasive film with a diamond coating, with the nominal grain size of 0.5 μm and its modified type, additionally treated with a graphite layer, were investigated. Chemical composition tests of these two types of surfaces with different manners of modification showed the same results, hence both of them showed a 100% carbon content. In the case of the treated film, this means that the carbon being detected had its origin not only from the diamond abrasive layer, but also from the deposited graphite coating (Figure 6). This confirmed that the material structure of both the conventional and modified films was homogeneous, which should lead to a homogeneous performance of the film in abrasive processes. Other added benefits of having a graphite top layer on the diamond-coated film included improved lubricating properties and better thermal stability—all without any deterioration of the structural integrity required by the film. All of these results are indicative of the good strength and flexibility of both standard and modified 0.5 DLF films. It is, however, noted that in applications where increased machine ability requires a combination of abrasive grit size and more surface treatment, like adding levels of graphite, that such films substantially enhance the performance and surface finish. Some important differences between the conventional abrasive film (Figure 7) and the one integrated with a graphite layer (Figure 8) were found during an examination of surface topography. The former had higher degrees of surface irregularity including more distinct peaks and valleys that were dispersed over the surface, while the latter presented a rather homogeneous surface with smoother features and reduced peak-to-valley height. The deviation observed was ascribed to the presence of a graphite coating that filled up the interstitial gaps and improved the surface homogeneity. There existed a more heterogeneous distribution of features in the morphology of the reference film, with higher contrasts between raised and depressed regions, which could be indicative of visible abrasive grains. On the other hand, one film with a graphite coating had quite a homogeneous topography—a clear sign that the graphite layer was well spread out at the surface and controlled the smooth visual appearance of the product. In conventional films, individual abrasive particles and their boundaries were clearly distinguished from each other; in many cases, exposed directly by the abrasive material. In the graphite-coated film, the boundaries of the particles were not as prominent because the graphite layer smoothed out the surface, making it more level.

**Figure 9:**
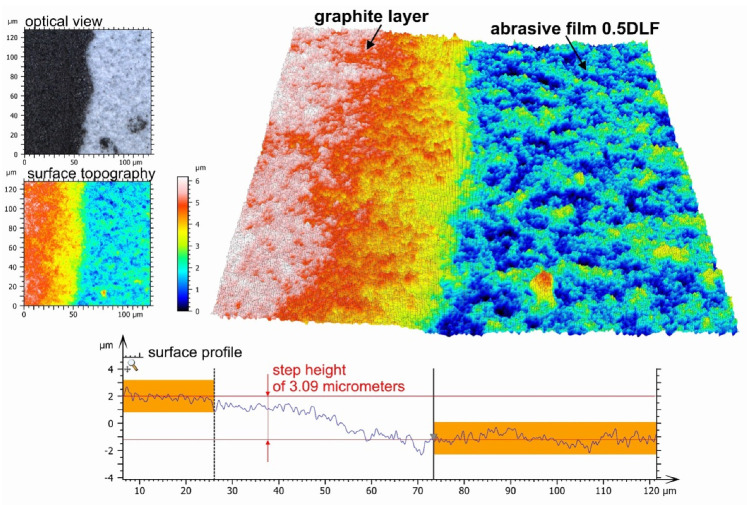
The 0.5DLF abrasive film coated with a graphite layer, along with the measurement of the graphite layer thickness (3.09 μm) determined from the surface profile at the boundary area between the carbon layer and the abrasive coating.

The thickness of the graphite layer was determined by analyzing the interface between the graphite coating and the abrasive surface. From the optical image of the surface, the graphite layer can be observed in black, while the diamond abrasive coating appears light blue in color. Likewise, this topographical map revealed huge differences between these two regions. One profile, aligned at a right angle to the edge of the graphite layer, was taken from the observed area. From the profile extracted, the average heights of the graphite layer and diamond-coated abrasive were calculated. The difference between the two averages gives the thickness of the graphite layer; for this sample, a thickness of 3.09 μm was found (Figure 9).

This provides a clear-cut image of the boundary, enabling the graphite coating to be quantified with precision. The optical image underlines the fact that there was a high contrast between the two materials, which means that the deposition of the graphite layer on an abrasive surface was performed effectively. Moreover, the topographic map confirms this point, since it illustrates the variation of height in the two regions, hence underlining the homogeneity of the graphite layer with respect to the bottom abrasive coating. The high accuracy of the measurement method applied proves the effectiveness of the deposition technique in creating a homogeneous and well-defined graphite layer.

### 2.2. Analysis of Finishing Results

**Figure 10:**
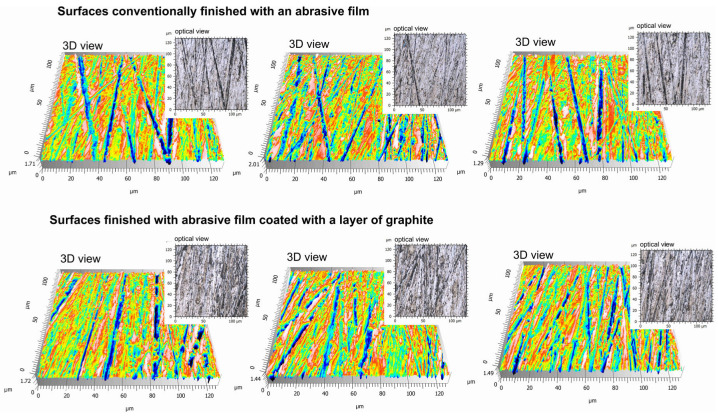
Surfaces machined with the conventional 0.5DLF abrasive film, processing time 60 s ((**top**) of the figure), and surfaces machined with the 0.5DLF abrasive film coated with a graphite layer, processing time 60 s ((**bottom**) of the figure). Each surface is presented as a topographic map and an optical image.

Titanium alloy specimens that had undergone identical preliminary machining operations were microfinished for one minute. One specimen was processed using a standard 0.5DLF abrasive film, while the second specimen was processed with the same film but with its surface coated with an approximately 3-micrometer-thick layer of graphite. Five measurements were made on each specimen, and Figure 10 shows three representative surfaces from each group. Optical imagery did not point out any meaningful differences in the surface appearance of both samples, which suggests that under the measurement conditions, and especially in the 126 × 126 μm measurement area, the graphite layer on the surface was highly transparent. Optical images obtained from both categories showed a very homogeneous structure, which allows for the conclusion that at this magnification, the graphite layer did not alter the surface characteristics in a meaningful way. Similarly, the 3D topographic views did not show outspoken differences between the samples. Both groups represented similar patterns of surface irregularities, and the occurrence of grooves and peaks appeared consistently through the observed areas. Whereas minor variations in depth and smoothness may have existed, they were not of a degree that would unequivocally distinguish the effects of the graphite coating in the 3D representation. This was expected under the hypothesis, since the presence of a graphite layer was actually mixed into the abrasive film without appreciably changing the macro-scale surface texture.

**Figure 11:**
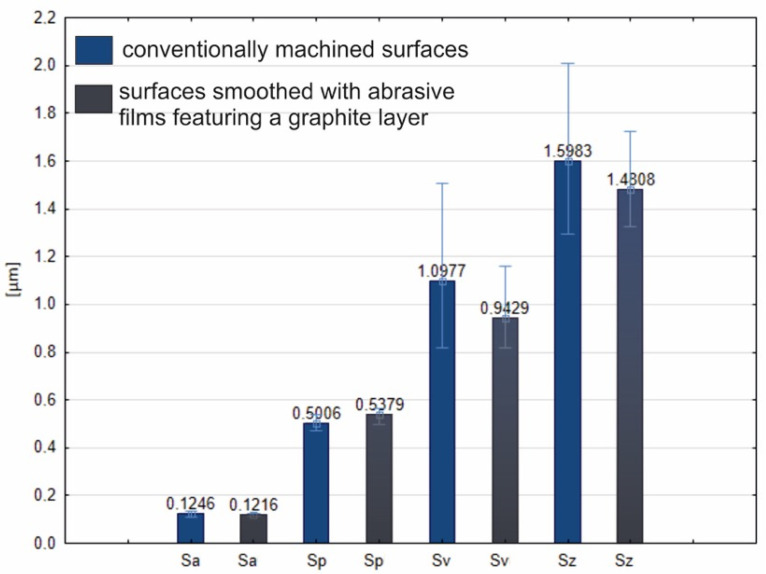
Surface roughness parameters determined from titanium samples machined conventionally (blue color) and using a tool with a graphite layer (gray color). The processing time in both cases was 60 s.

These findings suggest that the graphite layer contributed to the surface modification in a way not directly obvious from either the optical or 3D views under the given conditions. Thus, an in-depth analysis of the surface topography was carried out for both surfaces, and the results are shown in Figure 11, where the bars represent the measured parameters. Blue-colored bars symbolize the parameters for the surfaces treated with the traditional abrasive film, while gray-colored bars represent the surfaces finished with the graphite-coated film. Results regarding the Sa parameter were quite similar in both cases, with very slight differences between the five measurements for each sample, which suggests a high level of homogeneity. The differences encountered were at the nanometric level, with a slight advantage for the surface treated with the graphite-covered film. Slightly higher peak heights (Sp) could be observed on the graphite-coated film finished surfaces; however these differences were also still pretty minor. The largest difference was found for the Sv parameter, which describes the depth of the surface valleys or, for the present study, the depth of the machining grooves. This hints at the tendency of graphite to stick to the machining grooves and hence lead to the creation of a certain surface texture. Since the Sv parameter directly influences Sz, which is the total height difference between the highest peak and the lowest valley, this difference was also reflected in the Sz values. It can thus be stated that the graphite layer influences the surface texture by creating a more distinct pattern, since it deposits inside the machining traces. This would result in a texture, whose consequence in applications seeking a tailored surface property, for example, improved lubrication or resistance to wear, could be profound.

**Figure 12:**
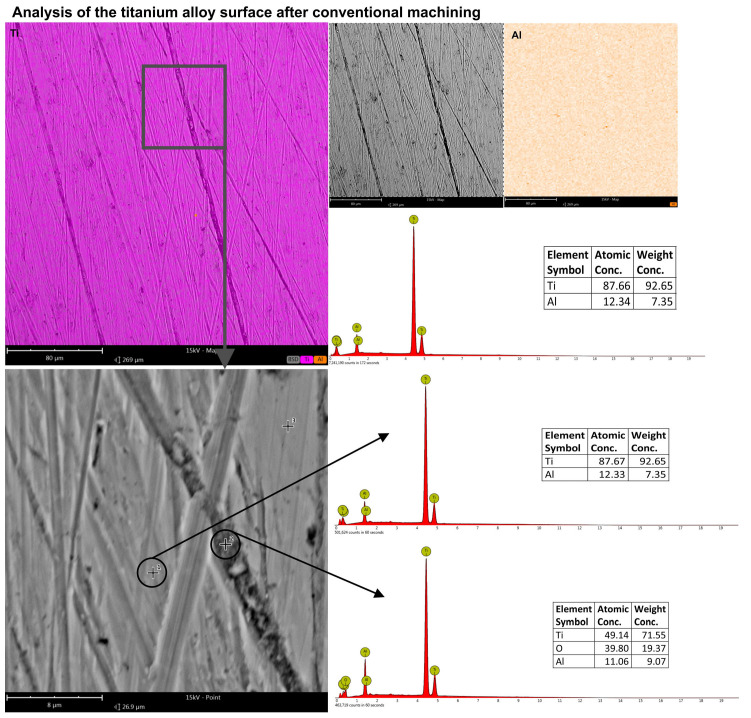
EDS analysis results of the titanium alloy surface smoothed conventionally, with measurements conducted on both the surface and at specific points along the machining trace as well as on the flat surface.

**Figure 13:**
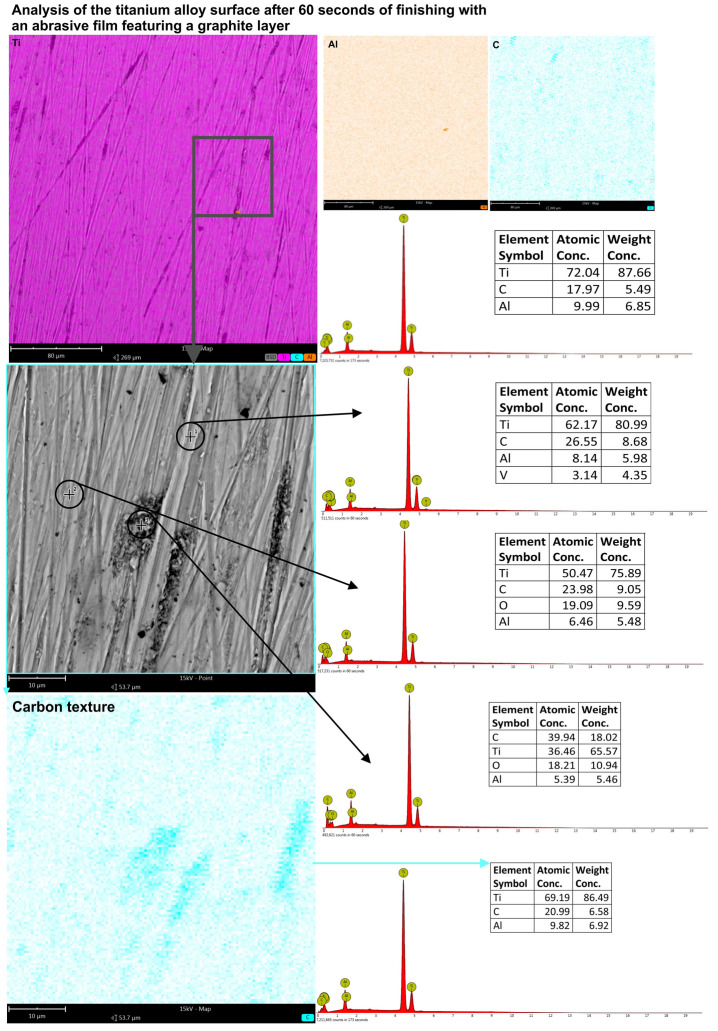
EDS analysis results of the titanium alloy surface smoothed using the abrasive film coated with a graphite layer, with a processing time of 60 s. Measurements were conducted on both the surface and at specific points along the machining trace as well as on the flat surface.

To establish that carbon, in particular, was deposited within the machining grooves, EDS analysis was conducted on such surfaces using both surface scan techniques and point-specific (spot) measurements. The study covered both conventionally machined specimens and those subjected to machining with graphite-coated abrasive films. For the conventionally machined sample, no detectable carbon was present in the analysis at high magnification and larger areas. The results were within the expected composition of the titanium alloy, showing approximately 87% titanium and 12% aluminum. In the machining grooves though, there were traces of the material that had been oxidized, as indicated by the additional presence of oxygen (Figure 12). In contrast, the sample treated by the graphite-coated abrasive film showed the presence of carbon on all parts of the whole surface, although in different amounts. A surface level EDS scan at a higher magnification showed approximately 18% carbon. More in-depth, point-specific analysis showed that the machining grooves contained 26.55% carbon, and smoother regions of the surface contained 23.98% carbon. In addition, small clusters of graphite accumulations were observed, containing as high as 40% carbon (Figure 13). It is clear from the results that a very short treatment, only 60 s, using the graphite-coated abrasive film led to the formation of a carbon layer on the processed surface. Hence, the assumption from this aspect would be that the increased carbon measured within the machining grooves may mean that abrasive action might improve the way that the graphite would deposit in the areas described and finally develop the peculiar surface texture characteristics. Accordingly, huge improvements within such aspects would take place during the surfacing of materials to acquire improved surface features for tribological functionality.

**Figure 14:**
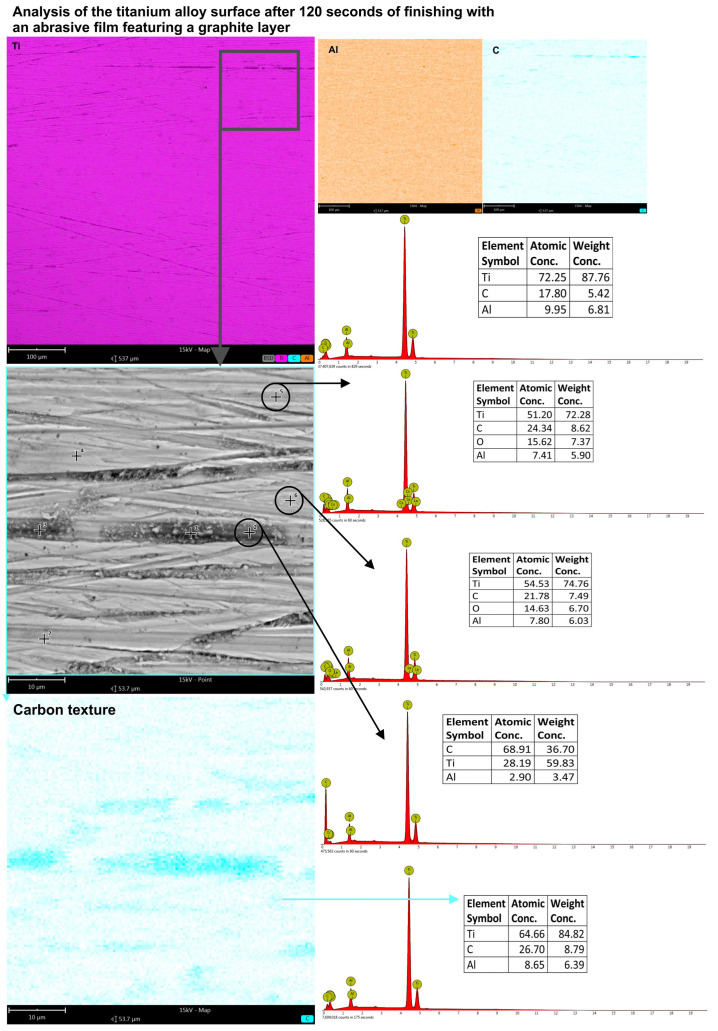
The results of the EDS analysis for the titanium alloy surface processed with a graphite-coated abrasive film, with a treatment duration of 120 s. The measurements were taken across both the surface and at designated points within the machining trace as well as on the flat regions.

In order to investigate the effect of microfinishing time on the formation of the carbon layer on the workpiece, the application of the graphite coating was continued. The data were collected after 120 s of processing and then after 240 s. The surface roughness and chemical composition of the polished surface were measured after each time gap. After 120 s, the alterations on the surface were not significantly different (Figure 14) in terms of the carbon composition on the treated surface. More specifically, at the maximum magnification on the surface examination by EDS, the carbon concentration was about 18% on the surface. In the more uniform areas of the sample, the point analysis indicated carbon concentrations of 24.34% and 21.78%, respectively. However, considering the carbon content measured at the surface, analysis at a higher magnification showed it to be 26.7% compared to 20.99% after 60 s, which is a huge difference.

**Figure 15:**
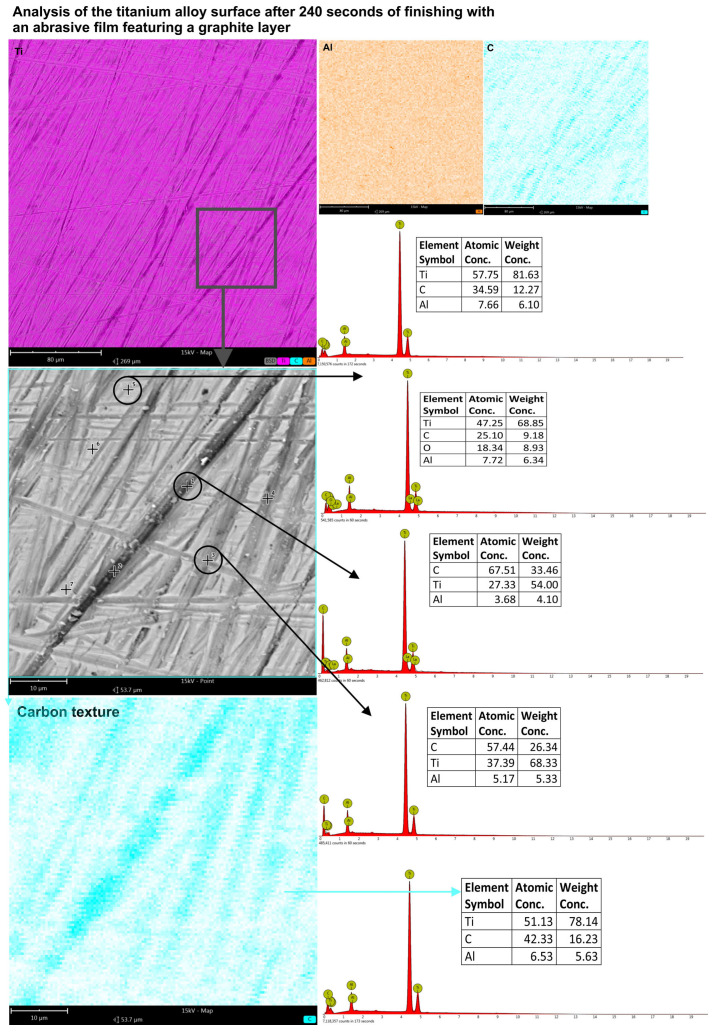
The EDS analysis outcomes for the titanium alloy surface treated with a graphite-coated abrasive film over a duration of 240 s. Measurements were performed both on the overall surface and at specific points within the machining path as well as on the flat areas.

The outcomes show that a longer processing time results in a higher carbon accumulation on the surface and especially in the machining grooves, which might favor the formation of a more uniform and dense graphite layer. The longer duration allows for a thicker deposition of graphite, hence it enhances tribological characteristics of the surface such as wear resistance and lubrication efficiency. Evidence of these changes became clearer with higher magnification and showed how important the optimization of the microfinishing process duration is in terms of controlling the thickness and homogeneity of the carbon layer. The most intensive carbon texture was obtained after 240 s of continuous microfinishing of the surface with the abrasive film with a graphite layer. The resulting texture was clearly visible in the EDS analysis results (Figure 15). The machining marks were well-defined and provide strong evidence for the fact that the highest accumulation of carbon took place inside the indentations of the machining grooves.

The carbon content on the scanned surface reached as high as 42.33%. In the deepest machining grooves, the carbon concentration even reached 67.51%. The results shed light on the strong influence of the graphite-coated abrasive film in changing the surface features during the microfinishing process. The deposition of carbon, most of which was located in the grooves, led to the formation of an individual texture that considerably increased the tribological properties of the surface such as good resistance to wear and lubricant performance. The results show that a longer period of microfinishing seems to favor the formation of a more obvious and thicker carbon layer, which would provide a better performance mainly for components requiring particular surface conditions. The innovations of surface texture and composition make the microfinishing process, using a graphite-coated abrasive film, highly effective in applications requiring high material performance.

**Figure 16:**
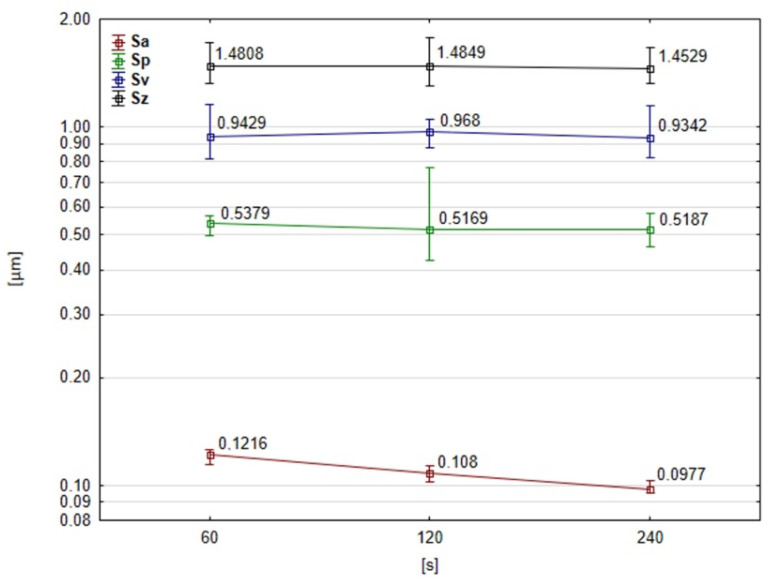
Parameters for evaluating the surface roughness with abrasive films coated with a graphite layer as a function of time.

The topographical features of the workpieces at five different positions were analyzed for each time period during the coupled microfinishing and carbon layer deposition processes. Figure 16 shows the roughness parameters of the treated surfaces. It can be seen that the depth of the grooves—represented by the parameter Sv—was not affected by the increase in processing time. This would imply that this increase in carbon level in the machining grooves was not due to a decrease in the depth of the grooves, and is thus evidence that the thickness of the carbon layer increased homogeneously and not just merely within the machining traces. The only parameter that significantly decreased with increasing processing time was the Sa parameter. However, this has less to do with the deposition method of the carbon layer and more to do with the intrinsic nature of the abrasive process. It was also noticed from the graph that the roughness of the surface, expressed as Sa, had decreased with time due to the smoothing effect, a characteristic attributed to the abrasive action. On the other hand, Sv kept a remarkably stable value, which supports the suggestion that increasing the carbon concentration in the grooves did not result in reducing their depth. It confirms the assumption that during the running-in process, the graphite layer became thicker, and this thickness provided the possibility of surface modification while maintaining the constant depth of the machining traces. The results from this study demonstrate that the most evident consequence of the deposition of this graphite layer was the improvement in the quality of the surface (i.e., its smoothness) and probably improved the tribological characteristics, while the depth of the grooves was hardly affected.

### 2.3. Analysis of Abrasive Film Surfaces After the Microfinishing Process

**Figure 17:**
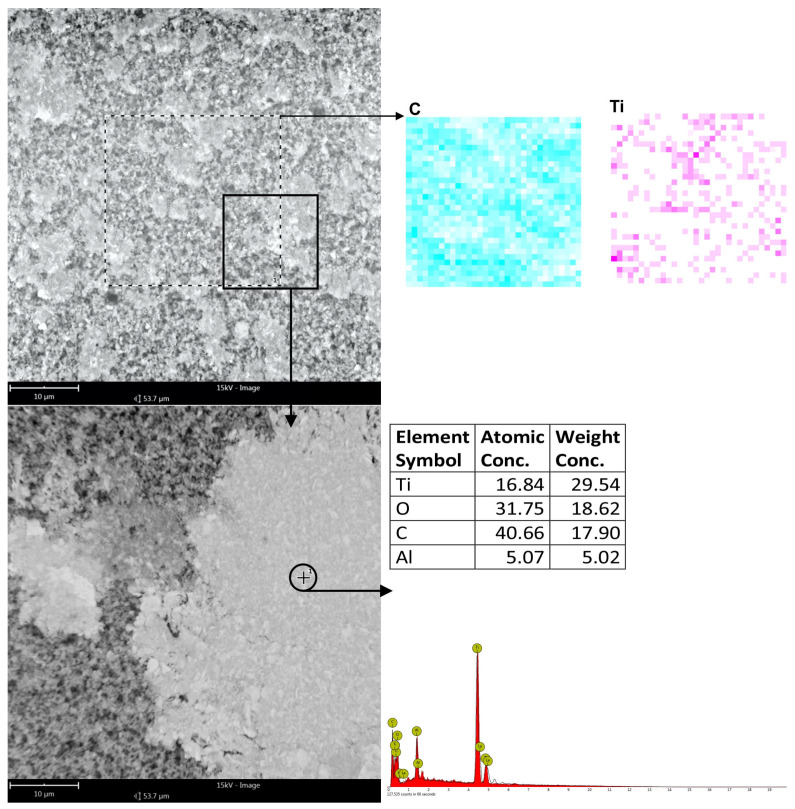
The surface of a conventional abrasive film after the microfinishing process, with machining residues in the spaces between the grains, accompanied by the chemical composition analysis.

**Figure 18:**
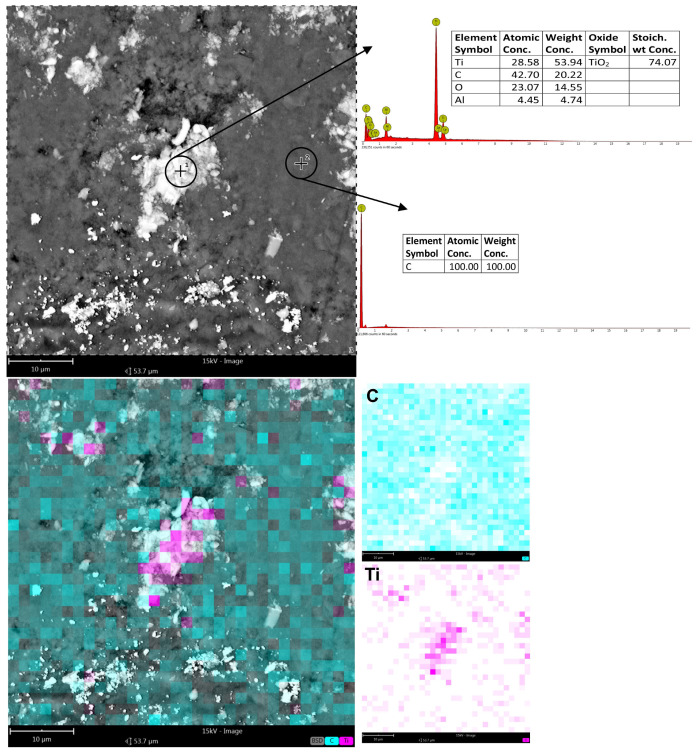
The surface of a graphite-coated abrasive film after the microfinishing process, with machining residues in the spaces between the grains, accompanied by the chemical composition analysis.

It is evident from the surfaces presented in Figure 17 and Figure 18, respectively, for the conventional abrasive film and the graphite-coated abrasive film, that after a microfinishing time of 60 s, some important observations can be made. In the case of the conventional abrasive film (Figure 17), worn machining products mixed together with binder and abrasive grains were recognized. This was confirmed by the chemical composition analysis of the surfaces, showing the presence of Ti, O, and Al in a normal ratio for the titanium alloys with a slight amount of oxygen, which suggests that some oxidized material had been left over on the surface after machining. The surface showed signs of degradation and interaction between the abrasive grains and the binder, leading to the formation of organized areas where the machining swarf had deposited in the channels, possibly leading to the formation of a more compact structure. On the other hand, the surface treated with the graphite-coated abrasive film (Figure 18) showed clear signs of graphite compression during the finishing process as well as clear marks of graphite compression during the finishing process. The surface, on casual observation, had a luster that was appreciably brighter than the matte finish initially observed prior to the microfinishing process. Compression of the graphite did not remove the voids caused by the machining by-products; instead, the titanium shavings appeared to be scattered in a much more random fashion, without the induced structured regions commonly seen in traditional abrasive films. This would increase the lubrication or cooling of the graphite layer during the process, yielding a more accurate surface and possibly lowering the temperature in the machining zone.

This temperature drop could be an indicator of a less harsh cutting process, which could lead to lower tool wear and a more uniform deposition of carbon on the surface. It can be concluded from the results that the use of a graphite-coated film during microfinishing played a vital role in the reduction in friction and an improvement in the overall surface quality for a better finish as well as a probable improvement in the tribological properties of the surface. These results further confirm the advantages of the use of a graphite-coated film, specifically in applications where desired surface characteristics with less wear are required.

## 3. Materials and Methods

The sample used was a Grade 5 titanium alloy, namely Ti–6Al–4V. The sample was prepared by a series of preprocessing steps involving abrasive films in decreasing order of nominal grain size: 30 to 12 to 9 to 5 to 3 to 1 micrometer. All of the above steps constituted the preparatory stage, and the actual experimental process made use of an abrasive film impregnated with diamond abrasives with a nominal grain size of 0.5 μm.

The preparation of the lapping film starts with the saturation of interstitial spaces between the abrasive particles by means of graphite. Preparation by this means ensures that the graphite is spread uniformly throughout the film. This film is coupled into the system at velocity (*v_t_*). As it does so, the film comes into contact with the pressure roll, which presses against the film with a force, (*F_r_*); it is, in effect, squeezing down on the surface of the workpiece. Under this pressure, the graphite can be evenly transferred onto the workpiece surface.

The workpiece, placed in the machining area, is rotated at a prescribed velocity (*v_w_*) for the uniform deposition of graphite over its entire surface. Concurrently, abrasive particles in the lapping film polish the workpiece surface and carry graphite to form a thin, uniform carbon layer. The interactions between the pressure roll and abrasive properties of the film, along with relative movement between the workpiece and the film itself, result in a machined surface with homogeneous carbon deposition. The coating process enhances the properties of a surface, reduces roughness, and increases the tribological performance, hence, it is widely accepted in many applications. The experimental equipment used in the carbon layer deposition process is shown in Figure 3. The standard parameters used for this microfinishing process are given in Table 1. Machining was conducted by using GW-1 (Koszalin, Poland), as shown in Figure 4, installed at an engine lathe from the cutter holder position. This attachment accepts tools of “1/2”, “1”, or “2” in width. Its tool feed rate varies from *v_f_* = 0 to 90 mm/min (extendable to 500 mm/min), while its oscillation frequency and amplitude are *f_0_* = 0 to 500/min and A = 2.5 mm, respectively. The roll pressure, controlled by a pneumatic actuator from a 0.6 MPa system, varies between *F_r_* = 10 and 90 N (up to 200 N). The attachment runs on a 230 V, 400 W power supply, measures 575 × 250 × 300 mm, and weighs 25 kg.

The first stage in this research was microfinishing for 60 s using a conventional abrasive film and a film with an applied graphite layer. Comparative analysis of the results obtained by both methods was performed to assess the effect of the graphite layer on the finishing process and surface characteristics. The surface topography of the titanium alloy was measured by an Olympus LEXT OLS4000 confocal microscope (Tokyo, Japan), which also acquired optical images of the carbon layers formed. The surfaces of the abrasive film and the processed surfaces were measured using an LMPLFLN100X lens (Tokyo, Japan) (with a magnification of 2160×, a field of view (FOV) of 128 μm, a working distance (WD) of 3.4 mm, and a numerical aperture (NA) of 0.8). For each processed surface, measurements were taken at five distinct locations. The following parameters were determined to assess the surface roughness of each sample. Calculations adhered to the ISO 25178 standard, particularly for the height-related measurements [57]:

Sa: the arithmetical mean height of the surface;
Sz: the maximum height of the surface;
Sp: the maximum peak height;
Sv: the maximum pit depth.

The results are presented as a chart, where the variability for each sample is depicted using min–max whiskers, while the displayed value represents the arithmetic mean of the five measurements. To measure the step height, corresponding to the thickness of the graphite layer on the film surface, a step height analysis was employed. For each step, the average value from a specified section of the profile was determined, and then the distance between these averages was calculated, representing the thickness of the deposited carbon layer.

Scanning electron microscopy (SEM) and energy-dispersive spectroscopy (EDS) imaging of the carbon layer surfaces was carried out by a Phenom ProX tabletop electron microscope from Phenom-World BV, Eindhoven, The Netherlands. For the chemical composition measurements, scanning was performed at an accelerating voltage of 15 kV, along with a point scan, following the manufacturer’s guidelines for the Phenom ProX system. This approach ensured accurate detection of the elemental composition, leveraging the system’s EDS capabilities to analyze localized areas with high precision.

## 4. Summary and Conclusions

This paper investigated the formation of carbon layers on the surface of the Grade 5 titanium alloy Ti–6Al–4V when subjected to a microfinishing operation using conventional abrasive films and graphite-coated abrasive films. The presented study is in line with a microfinishing operation intending to produce an improved quality surface in order to improve the tribological properties of the titanium alloy. The process used was founded on diamond-particle-infused abrasive films, further modified by a graphite coating to reduce the surface roughness while improving the efficiency. Microfinishing was an effective method in reducing the surface roughness, first by creating a uniform carbon layer that was coated on the titanium alloy as a result of the contact between the abrasive film and the pressure roll and the relative motion between the workpiece and abrasive film. The resulting carbon layer is an important factor that increases the surface lubricating and thermal properties, with many applications in the field of wear resistance and friction reduction. EDS analysis underlined that the graphite coating of the abrasive film ensured the homogeneous distribution of carbon over the treated surfaces. Hence, an opportunity was presented to adjust the surface texture on the mentioned coatings for quite a number of industrial purposes.

- The inclusion of a graphite coating on abrasive films significantly enhanced the surface finish quality; it generated a uniform layer of carbon and therefore enhanced the tribological features of the surface.
- The use of graphite-coated abrasive films provided smoother surface finishes and resulted in more a homogeneous carbon distribution. This proves that the application of the carbon layer did not change much in the depth of the machining grooves on the surface, proving that the thickness of the graphite film increased uniformly over the whole surface.
- More extended processing times resulted in thicker carbon layers, thus increasing the wear resistance and improving the thermal stability, which are beneficial in applications where specific surface properties are important.
- The results showed very the high potential of graphite-coated abrasive films for the improvement of surface quality, mainly when there are requirements for components subjected to high lubrication, wear resistance, and surface integrity.

## 5. Patents

W. Kacalak, K. Tandecka, B. Bałasz, K. Rokosz: Method for Producing Carbon Nanolayers on Surfaces at the Time of Micro-Smoothing Them with Abrasive Films. Patent No. Pat.240472, filed on 17 April 2018, granted on 17 January 2022, valid until 17 April 2038. Application No. P.425253. IPC Classification: B82Y 30/00, B24D 11/02, B24D 3/00, B32B 7/10.

## Figures and Tables

**Table 1:** Conditions for the experimental machining.

Workpiece Material	Pressure Roll Hardness	Pressure Force	Tool Speed	Workpiece Speed	Oscillation Frequency	Processing Time
Titanium Alloy Grade 5 (Ti–6Al–4V)	5° Sh	50 N	160 mm/min	10 m/min	80 Hz	60, 120, 240 s

## Data Availability

Data are contained within the article.
